# Surgical treatment of meningiomas improves neurocognitive functioning and quality of life – a prospective single-center study

**DOI:** 10.1007/s00701-024-06295-5

**Published:** 2024-10-10

**Authors:** Moritz Ueberschaer, Rene Hackstock, Lucas Rainer, Katharina Breitkopf, Arwin Rezai, Andreas Kaiser, Christoph J. Griessenauer, Christoph Schwartz

**Affiliations:** 1 Department of Neurosurgery, University Hospital Salzburg, Paracelsus Medical University, Salzburg, Austria; 2 Institut for Clinical Psychology, Department of Psychiatry, Psychotherapy and Psychosomatics, University Hospital Salzburg, Paracelsus Medical University, Salzburg, Austria; 3 Department of Child and Adolescent Psychiatry, Christian Doppler University Hospital, Paracelsus Medical University, Salzburg, Austria; 4 Department of Neurology, Christian Doppler University Hospital, Paracelsus Medical University, Member of the European Reference Network EpiCARE, Salzburg, Austria

**Keywords:** Meningioma, Neurocognitive testing, Neuropsychology, Quality of life, Depression

## Abstract

**Background and purpose:**

Early diagnosis and the refinement of treatment of patients with intracranial meningiomas have brought quality of life (QoL) and neurocognitive functioning as outcome measures into focus. The aim of this study is a comprehensive assessment of neurocognitive function, quality of life and the presence of depression in meningioma patients before and after surgery.

**Methods:**

Patients with MRI diagnosis of intracranial meningioma and indication for surgery were prospectively included. A clinical neuropsychologist performed neurocognitive assessments within 3 months before and 12 months after surgery. The test battery included investigation of selective and divided attention, verbal and figural memory, executive functioning, and word fluency. Self-report questionnaires to assess depressive symptoms, QoL, and disease coping were administered. Raw values and t-values were compared pre-and postoperatively. Outcome was stratified by tumor- and peritumoral brain edema (PTBE) volumes, postoperative resolution of PTBE and WHO grade. The study included 18 predominantly female patients (83%) with a median age of 59 years and mostly CNS WHO grade 1 meningiomas (83%).

**Results:**

There was a significant postoperative improvement in the ability to selectively react under stress, in working memory and improved delayed reproduction of verbal and visual memory content. QoL improved regarding a reduction in physical problems, an improvement in energy, and social functioning. There was a trend towards worse preoperative scores in all tests, and greater postoperative improvement in patients with PTBE. Tumor volume had no effect on the measured outcome. The patients did not suffer from depressive symptoms before the surgery but improved postoperatively and most patients had an active, problem-oriented coping strategy.

**Conclusion:**

Resection of intracranial meningiomas leads to an improvement in multiple neurocognitive domains and QoL. There is a trend towards poorer preoperative neurocognitive functioning and greater postoperative improvement in patients with PTBE. Depression appears to play a minor role in the context of neurocognitive functioning and disease coping.

## Introduction

 Meningiomas are the most diagnosed intracranial tumor entity [21]. Histopathological grading is based on histological and molecular criteria in accordance with the 2021 World Health Organization (WHO) classification, and the vast majority of meningiomas are of benign nature (WHO grade 1) [15]. Small meningiomas usually only require regular follow-up imaging [25]; however, treatment is indicated in case of tumor growth, space-occupying effects, and/or symptomatic lesions [8]. In most cases, the treatment of choice is microsurgical tumor resection with or without radiotherapy, depending on the extent of resection and tumor grade. Tumor resection is usually associated with low morbidity and tumor control rates of up to 88% have been reported for benign meningiomas [23]. The predominantly good prognosis and good overall neurological function after surgery lead to a particular interest in more complex neurological functions such as neurocognition and neuropsychological aspects, which contribute significantly to patients’ quality of life (QoL) [28].

Previous studies suggested that small, incidentally diagnosed meningiomas are “cognitively benign” tumors, meaning that they usually do not affect the patients’ neurocognitive functions [8]. In contrast, meningiomas with mass effect and/or peritumoral edema that warrant treatment may cause neurocognitive impairment, and prior studies suggest that surgery may lead to improvement [12, 17, 25, 26, 32]. Hendrix et al. [9] prospectively examined 12 patients with frontal meningiomas and found pre- and postoperative deficits in perceptual speech, executive function, short-term memory and verbal fluency with postoperative improvement in executive function and short-term memory. Di Cristoferi et al. [4] prospectively studied elderly patients (> 70years) preoperatively and at 3 and 12 months postoperatively. They found preoperative neurocognitive deficits in over 97% of their cohort and demonstrated that neurocognitive improvement was especially evident after the 12 months test. The largest study by Rijnen et al. [22] included 219 patients with pre- and postoperative tests at 3 months of which 89 patients were also tested at 12 months. The results are in line with the other cited studies confirming preoperative poor neurocognitive function that improves postoperatively but remains under average according to the calculated z-scores. However, many studies have several limitations, such as the lack of preoperative testing, the use of tests that do not allow a comprehensive assessment of neurocognitive function, or the inclusion of patients who have undergone additional radiotherapy [15]. Therefore, a recently published comprehensive systematic review called for more prospective studies to overcome these various limitations [6]. We conducted a comprehensive battery of neuropsychological tests in patients with surgically treated meningiomas before and after surgery to investigate the outcome in various neurocognitive domains. As QoL seems to be influenced by the neurocognitive function [19], we also included QoL and the aspects of depression and disease coping.

## Methods

All patients older than 18 years of age with imaging diagnosis of an intracranial meningioma and an indication for surgery were prospectively included over a period of 4 years. Patients with previous neurocognitive impairment for other reasons were excluded. Informed consent was obtained by all study participants. Initially, a consecutive inclusion of patients in the study was planned. However, many patients (36/44; 82%) did not show up for postoperative testing, mainly because they did not want to undergo the time-consuming and complex tests. Demographic data including age, sex, previous diseases, and symptoms were documented (Table 1). Prior to the final data analysis, the last follow-up including tumor recurrence and time to recurrence was evaluated. The study was approved by the local ethics committee (415-E/2122/7-2017).

**Table 1:** Demographic data and tumor characteristics of the patient cohort

	*n* = 18
Age years (range)	59 (33–75)
Female Sex (percentage)	15 (83%)
Follow up (months)	61.1 ± 26.1
Previous diagnosis Arterial hypertension Hypothyroidism Diabetes Others	9/18 (50%) 6 2 2 4
Time until test before surgery (days)	10 ± 18.2
Time until test after surgery (days)	190.6 ± 34.7
Tumor location Skull base Falx Convexity Frontal Temporal Parietal Infratentorial Right Left Bilateral	8 6 4 9 4 3 2 6 11 1
Tumor Volume (cm^3^)	27.0 ± 37.3
Edema Volume (cm^3^)	8/18 (44%) 26.0 ± 23.5
Histopathology WHO grade 1 WHO grade 2 WHO grade 3	15 2 1
Postoperative complications (wound healing disorder)	2/18 (11%)
Tumor remnant on postoperative MRI	3/18 (17%)
Postoperative radiotherapy (after test)	2/18 (11%)
Tumor recurrence Time to recurrence (months)	3/18 (17%) 45.3 ± 23.9

Either mean values ± standard deviation or median (range) are given

### Patient cohort

Included were 18 predominantly female (83%) patients with intracranial meningiomas. Fifteen (15/18) patients had WHO grade 1, 2/18 grade 2, and 1/18 grade 3 tumors. One tumor had a borderline grade 1/2 characteristic with a Ki67 index of 30% and was therefore categorized as a grade 2 tumor. None of the grade 2 tumors were categorized as grade 2 solely on the basis of brain invasion. Most tumors (*n* = 11) were lateralized to the left hemisphere, and the most common tumor locations were the frontal convexity (*n* = 9) and the skullbase (*n* = 8). The mean tumor and PTBE volumes were 27.0 ± 37.3 cm^3^ and 26.0 ± 23.5 cm^3^, respectively. The most common signs or symptoms to trigger initial cerebral imaging were headaches (*n* = 5) and epilepsy (*n* = 4). Tumor remnants defined as residual solid contrast enhancement in the area of tumor resection according to postoperative MRI were seen in 3/18 (17%) patients (one WHO grade 2 and two grade 1 tumors). 1/18 (6%) patients with WHO grade 3 tumor received adjuvant radiotherapy and 1/18 (6%) patients with a WHO grade 2 tumor received radiotherapy for tumor recurrence. Of note, postoperative neurocognitive testing was always performed before radiotherapy (88 days and 27 months). Within a mean follow-up of 61.1 ± 26.1 months, tumor progression, occurred in 2 patients with postoperative tumor residual (one WHO grade 1 and one grade 2 tumor) on MRI and tumor recurrence was found in 1 patient (WHO grade 1 tumor) without postoperative residual tumor after a mean time of 45.3 ± 23.9 months after surgery (3/18 (17%)). All demographic data are shown in Table 1.

### Neuropsychological assessment

The selection of tests aimed at a comprehensive assessment of the various neurocognitive domains. A combination of computerized and paper-based tests was chosen. For each test, raw values and t-values, which are calculated to compare the individual patient’s test result with an average population (values of 40 to 60), were determined within 3 months before surgery and within 12 months after surgery. In fact, the preoperative tests were performed 10 ± 18.2 days before surgery and the postoperative tests 190.6 ± 34.7 days after tumor resection. The time interval was chosen to reduce the likelihood of a learning effect and to take into account the results of Di Cristoferi et al. [4] who showed significant improvements in various neurocognitive domains at a 12-month postoperative test interval compared to a 3-month test. All tests were conducted by a certified clinical neuropsychologist (R.H.). All neurocognitive tests provide representative norm samples adjusted for age, gender and education. The tests are also available in English.

#### Determination test

The Determination Test (DT) is a computer based test from the Vienna Test System [24] to evaluate selective attention and reactivity. The test is an accurate procedure for measuring reactive resilience and the associated reaction capability. The participant is presented with color stimuli and acoustic signals. The response is made by pressing the corresponding keys on the participant’s keyboard.

#### Wechsler memory scale IV

The Wechsler Memory Scale IV (WMS-IV) [30] is a neuropsychological instrument to assess different domains of memory functions. The subtests on verbal and visual memory were used. Verbal (logical) memory is assessed with an immediate and delayed recall of two verbal presented stories. Visual memory is assessed with an immediate and delayed recall, as well as recognition of shortly (10 s) presented figures.

#### N-back verbal test

The N-back verbal test (NBV) is a computer based test from the Vienna Test System [24]. The test captures verbal working memory. One of the most important tasks of working memory is maintaining and updating verbal contents (visual contents are implemented with the N-back nonverbal test). The test procedure implements the N-Back paradigm. The test requires both (a) a specific function of verbal working memory, the updating of contents, as well as (b) operationalizing the performance capability of verbal working memory “overall.” Disturbances in working memory play a central role in a variety of clinically relevant disorders or injuries, for example in connection with Alzheimer’s, Parkinson’s, or ADHD. Therefore, this procedure is mainly used in clinical neuropsychology, measuring working memory as part of the executive functions.

#### Wechsler adult intelligence scale IV

The Wechsler Adult Intelligence Scale IV (WAIS IV) [5] is an intelligence quotient test measuring various cognitive abilities in adults and adolescents. In total comprising ten subtests, we used the subtest symbol search and matrix reasoning (standard progressive matrices (SPM)), investigating processing speed and nonverbal abstract problem-solving / inductive reasoning, respectively.

#### Verbal fluency test

The Regensburger Wortflüssigkeitstest (RWT) [1] assesses verbal fluency. Specifically, we measured semantic fluency by counting the number of animals and the number of – alternating – fruits and sports, as well as lexical fluency of words starting with the letter “S” and, again alternating, words starting with “G” and “R.” For each condition, the participant was given 2 min time.

### Self-report questionnaires

#### Quality of life

Quality of lfe was assessed using the 36 Health Survey (SF-36) [29]. The SF-36 is a questionnaire on QoL with 36 items covering eight different domains of life.

#### Beck depression inventory II

The Beck Depression Inventory II (BDI II) [2] is a well-known self-report questionnaire to measure depressive symptoms and suicidality.

#### Freiburg questionnaire on coping with disease

The Freiburg Questionnaire on Coping with Disease (FQCD) [19] is a self-report questionnaire with 35 items measuring depressive coping, active coping, distraction/self-affirmation, search for meaning, and cognitive avoidance/dissimulation.

### Surgery and imaging analysis

Microsurgical tumor resection was performed in a standardized fashion with the use of intraoperative neuronavigation and ultrasound. Resection aimed for maximum safe resection. Tumor volume was measured in preoperative contrast enhanced T1 MRI sequences and peritumoral brain edema (PTBE) volume was measured in preoperative T2 or flair MRI sequences using the SmartBrush tool of Elements Brainlab software (Brainlab, Munich, Germany). Postoperative MRI was evaluated for tumor residuals on T1 with contrast enhancement and FLAIR sequences. In patients with PTBE, the postoperative MRI was also evaluated for resolution of PTBE. In the case of persistent PTBE, a volumetric analysis was performed in the same way as preoperatively. The median time between postoperative MRI and neurocognitive testing was 21 days (range 2-217days).

### Statistical analysis

Statistical analysis was performed using SPSS Statistics (version 29.0.2.0, IBM Corp.). For normal distributed numbers, the mean value (± standard deviation) was given. If data were scattered, the median (range) was given. A comparison of pre- and postoperative raw values was performed using the Mann-Whitney U-test for nonparametric variables. A significance level of *p* < 0.05 was set to determine statistical significance. Because of the small sample size, no further subgroup analyses were performed. For the definition of large and small tumors, the mean value of the entire cohort was chosen as the cut-off.

## Results

### Neurocognitive tests

The correct answers in the Determination Test increased significantly postoperatively, which indicates an improvement in the ability to react under stress. The Wechsler Memory Scale showed postoperative improvements in all subtests. Significant improvement was also reached in delayed logical memory reproduction (*p* < 0.001), logical verbal memory recognition (*p* = 0.008), delayed visual reproduction (*p* < 0.001), and recognition (*p* = 0.015). Working memory improved significantly after the surgery, as shown by the significantly higher values for correct answers in the N-back verbal (*p* = 0.043) and the lower values for missed answers (*p* = 0.043). Semantic verbal fluency assessed by the Regensburger test improved as well (*p* = 0.02). However, the other categories improved in absolute numbers without reaching significance. The Wechsler Adult Intelligence Scale IV symbol search and Progressive Standard Matrices showed only minor improvements. All mean values for the pre- and postoperative test results are listed in Table 2. The improvements were also reflected in the calculated T-values. However, the pre- and postoperative test results always ranged on average (40–60). Table 3 shows pre- and postoperative T-values.

**Table 2:** Comparison of preoperative and postoperative values for the neuropsychological test battery and self-report measures

Test	Preoperative	Postoperative	*p*-value
Determination Test (*n* = 17)			
Correct answers	195.35 ± 53.97	214.71 ± 41.19	**0.046**
False answers	8.12 ± 9.72	6.71 ± 4.52	0.6
Missed answers	14.71 ± 8.74	12.06 ± 8.77	0.13
Wechsler memory scale IV (*n* = 18)			
Logical memory direct reproduction	27.61 ± 6.81	30.72 ± 6.77	0.066
Logical memory delayed reproduction	21.39 ± 5.02	26.72 ± 6.72	**< 0.001**
Logical memory recognition	24.17 ± 2.81	25.94 ± 2.96	**0.008**
Direct visual reproduction	32.61 ± 5.57	33.17 ± 5.53	0.795
Delayed visual reproduction	19.83 ± 8.87	27.83 ± 8.08	**< 0.001**
Recognition	5.11 ± 1.78	6.00 ± 1.24	**0.015**
N-back verbal (*n* = 17)			
Correct answers	11.47 ± 2.65	13.06 ± 2.08	**0.043**
False answers	8.41 ± 12.71	6.76 ± 6.37	0.607
Missed answers	3.53 ± 2.65	1.94 ± 2.08	**0.043**
Wechsler Adult Intelligence Scale IV (*n* = 18)			
Symbol search	25.44 ± 9.02	28.44 ± 11.04	0.126
Standard Progressive Matrices (*n* = 18)			
Correct answers	15.78 ± 5.52	17.28 ± 4.99	0.073
Verbal fluency test (Regensburger Wortflüssigkeitstest (RWT)) (*n* = 18)			
Semantics (“animals”)	28.44 ± 10.83	32.78 ± 9.71	**0.020**
Semantic alternating (“fruits/sports”)	19.28 ± 6.46	21.28 ± 5.29	0.417
Phonematics (“s”)	19.22 ± 8.43	19.89 ± 6.79	0.647
Phonematics alternating (“G/R”)	16.50 ± 7.35	18.89 ± 5.60	0.083
Short Form-36 Health Survey (*n* = 16)			
General health	59.44 ± 13.16	67.47 ± 19.11	0.124
Role limitations due to physical problems	50.46 ± 41.76	20.29 ± 26.54	**0.025**
Role limitations due to emotional problems	24.88 ± 37.95	18.12 ± 30.06	0.156
Energy/fatigue	46.28 ± 22.15	60.19 ± 13.87	**0.030**
Emotional well-being	68.94 ± 15.91	78.25 ± 14.60	0.064
Social functioning	65.44 ± 22.33	80.47 ± 20.40	**0.049**
Physical functioning	80.41 ± 18.97	86.87 ± 13.02	0.130
Pain	19.85 ± 25.44	21.56 ± 34.95	0.964
Beck depression Inventory II (*n* = 14)	7.79 ± 3.31	4.86 ± 3.74	**0.042**

Comparison of pre- and postoperative values using the Wilcoxon test. Mean values ± standard deviation are given. Significant *p*-values are highlighted in bold

**Table 3:** Comparison of preoperative and postoperative t-values for the neuropsychological test battery

Test	Preoperative	Postoperative
Determination Test (*n* = 17)		
Correct answers	44.00 ± 10.94	49.53 ± 10.16
False answers	54.35 ± 12.29	53.94 ± 7.39
Missed answers	46.35 ± 11.27	51.12 ± 11.29
Wechsler memory scale IV (*n* = 18)		
Logical memory direct reproduction	46.11 ± 8.20	50.83 ± 9.99
Logical memory delayed reproduction	43.61 ± 6.78	51.11 ± 8.87
Logical memory recognition	48.44 ± 6.19	53.17 ± 4.58
Direct visual reproduction	45.56 ± 9.37	47.06 ± 8.89
Delayed visual reproduction	41.61 ± 9.08	51.22 ± 7.90
Recognition	46.28 ± 10.58	51.83 ± 7.45
N-back verbal (*n* = 17)		
Correct answers	47.76 ± 6.15	51.94 ± 5.76
False answers	47.88 ± 9.61	47.00 ± 8.17
Missed answers	48.59 ± 7.55	53.59 ± 7.45
Wechsler Adult Intelligence Scale IV (*n* = 18)		
Symbol search	47.11 ± 9.08	49.94 ± 11.81
Standard Progressive Matrices (*n* = 18)		
Correct answers	46.61 ± 7.91	49.67 ± 6.60
Verbal fluency test (Regensburger Wortflüssigkeitstest (RWT)) (*n* = 18)		
Semantics (“animals”)	46.33 ± 9.11	50.56 ± 9.80
Semantic alternating (“fruits/sports”)	49.00 ± 8.66	51.67 ± 10.19
Phonematics (“s”)	46.28 ± 10.71	48.72 ± 8.88
Phonematics alternating (“G/R”)	44.67 ± 10.11	48.00 ± 8.99

Mean values ± standard deviation are given

### Self-report questionnaires

According to the SF-36 questionnaire, patients stated less role limitations due to physical problems (*p* = 0.025), more energy (*p* = 0.03), and improved social functioning (*p* = 0.049) postoperatively. The most common method of coping with the disease was active problem-oriented coping. This includes actively seeking information about the disease and its treatments, making deliberate efforts to resolve problems, and creating and following a well-structured plan. It also involves a commitment to living more fully and a determined stance to cope with the illness. A depressive disease coping pattern was the least common (Table 4). With values below 8, the patients suffered neither pre- nor postoperatively from depressive symptoms according to BDI II. After the surgery, however, the values decreased significantly (7.79 ± 3.31 vs. 4.86 ± 3.74; *p* = 0.04).

**Table 4:** Freiburg questionnaire on coping with disease (*n* = 16)

	Mean values ± standard deviation
Depression processing	1.7 ± 0.59
Active problem-oriented coping	3.71 ± 1.01
Distraction and self-assembly	3.20 ± 0.92
Religiosity and search for meaning	3.00 ± 0.83
Trivialization and wishful thinking	1.72 ± 0.69

### The impact of PTBE, tumor volume and WHO grade on neurocognitive outcome and QoL

**Fig. 1:**
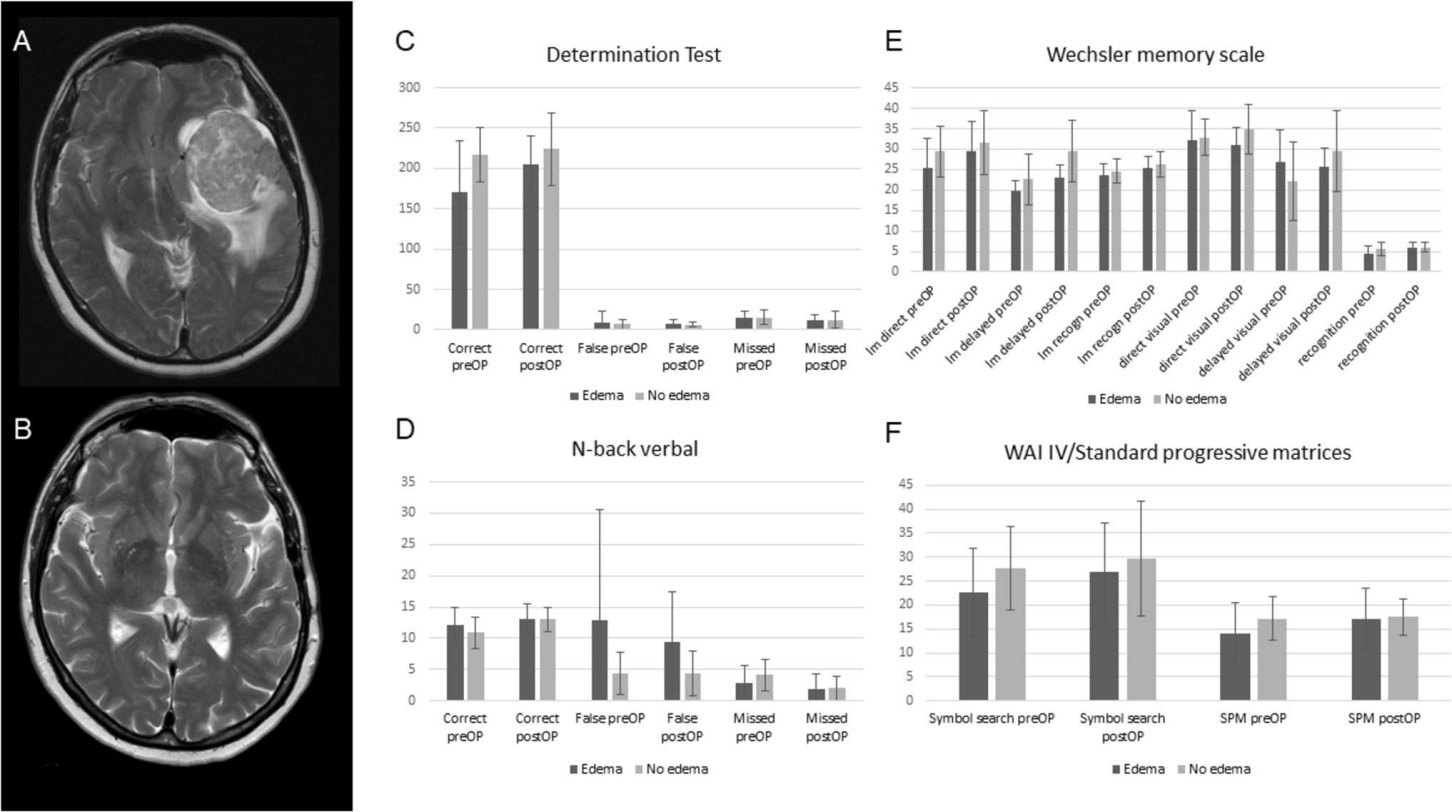
A preoperative T2-weighted MRI with a left temporal meningioma and PTBE; B postoperative T2-weighted MRI 3 months after resection of the tumor and completely resolved PTBE; C,D, E and F show results of different tests of patients with (blue) and without (orange) PTBE, reflecting the trend towards worse preoperative test results and greater postoperative improvement in the PTBE group

**Fig. 2:**
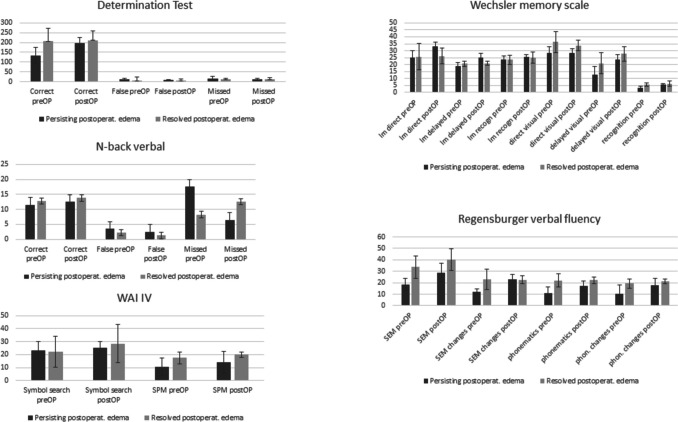
Comparison of pre- and postoperative test results, stratified by resolution (*n* = 4) or persistence (*n* = 4) of postoperative PTBE

The comparison of the test results in patients with (*n* = 8) and without (*n* = 10) PTBE showed a lower preoperative performance of patients with PTBE and a greater postoperative improvement in absolute numbers (Fig. 1). However, apart from the delayed reproduction of logical memory, no statistical significance could be demonstrated. In 4/8 patients PTBE resolved completely after surgery, overall the edema volume decreased from 26.03 cm^3^ preoperatively to 5.13 cm^3^ postoperatively. A comparison of the test results for DT, NBV, SPM, RWT and the visual subtest of the WMS of patients with and without resolution of PTBE shows a trend towards poorer preoperative and postoperative performance in patients with postoperatively persistent PTBE. In contrast, the WAI symbol search and the logical memory subtest revealed no difference between the two groups (Fig. 2). Tumor size > 27cm^3^ neither seemed to impact the neurocognitive function preoperatively nor postoperative improvement.

The comparison of the test results of 15 patients with WHO grade 1 and 3 patients with higher-grade tumors revealed poorer preoperative performance in the DT and logical memory subtests of the WMS, a higher rate of incorrect and missed responses in the NBV and poorer results in the RWF test in patients with higher-grade meningiomas. Postoperative performance improved primarily in patients with higher-grade meningiomas, with the scores of both groups equalizing. Only RWT and NBV performance remained worse in patients with higher-grade tumors (Table 5). Notably, the only patient in the cohort who had an aphasic component had a WHO grade 2 meningioma.

**Table 5:** Raw values of pre- and postoperative tests stratified according to WHO grade. *preoperative NBV and DT were missing in one patient

Test	WHO grade 1 (*n* = 15*)	WHO grade 2/3 (*n* = 3)
Determination Test (*n* = 17) preoperative		
Correct answers	201.86 ± 49.54	165.00 ± 75.36
False answers	5.50 ± 3.92	20.33 ± 19.60
Missed answers	14.71 ± 9.16	14.67 ± 8.08
Determination Test (*n* = 17) postoperative		
Correct answers	213.43 ± 45.33	220.67 ± 12.34
False answers	6.00 ± 2.69	10.00 ± 9.85
Missed answers	12.14 ± 9.61	11.67 ± 3.79
Wechsler memory scale (*n* = 18) preoperative		
logical memory direct reproduction	29.40 ± 5.68	18.67 ± 5.03
logical memory delayed reproduction	22.33 ± 4.98	16.67 ± 0.58
logical memory recognition	24.13 ± 3.04	24.33 ± 1.53
direct visual reproduction	33.13 ± 5.34	30.00 ± 7.21
delayed visual reproduction	19.73 ± 8.38	20.33 ± 13.32
recognition	5.13 ± 1.68	5.00 ± 2.65
Wechsler memory scale (*n* = 18) postoperative		
logical memory direct reproduction	30.80 ± 7.32	30.33 ± 3.79
logical memory delayed reproduction	26.73 ± 7.31	26.67 ± 3.06
logical memory recognition	25.73 ± 3.20	27.00 ± 1.00
direct visual reproduction	33.87 ± 5.36	29.67 ± 6.11
delayed visual reproduction	28.47 ± 8.62	24.67 ± 4.16
recognition	6.20 ± 1.08	5.00 ± 1.73
N-back verbal (*n* = 17) preoperative		
Correct answers	11.71 ± 2.70	10.33 ± 2.52
False answers	4.29 ± 3.75	27.67 ± 22.94
Missed answers	3.29 ± 2.70	4.67 ± 2.52
N-back verbal (*n* = 17) postoperative		
Correct answers	13.50 ± 1.74	11.00 ± 2.65
False answers	5.43 ± 4.97	13.00 ± 9.64
Missed answers	1.50 ± 1.74	4.00 ± 2.65
Wechsler Adult Intelligence Scale (*n* = 18)		
Icon search preoperative	25.80 ± 9.66	23.67 ± 5.77
Icon search postoperative	28.87 ± 11.77	26.33 ± 7.57
Standard Progressive Matrices (*n* = 18)		
Correct answers preoperative	16.00 ± 5.33	14.67 ± 7.57
Correct answers postoperative	17.27 ± 5.39	17.33 ± 2.89
Regensburger verbal fluency test preoperative (*n* = 18)		
Semantics	30.20 ± 9.50	19.67 ± 15.04
Semantic changes	20.60 ± 5.17	12.67 ± 9.45
Phonematics	19.93 ± 8.38	15.67 ± 9.50
Phonematic changes	17.13 ± 7.34	13.33 ± 8.02
Regensburger verbal fluency test postoperative (*n* = 18)		
Semantics	34.20 ± 9.37	25.67 ± 9.81
Semantic changes	22.00 ± 5.20	17.67 ± 5.03
Phonematics	19.93 ± 8.38	15.67 ± 9.50
Phonematic changes	18.87 ± 6.16	19.00 ± 1.00

## Discussion

### Key results

In this cohort, neurocognitive function improved in several domains as well as quality of life after meningioma surgery. Even though patients also showed a relatively lower BDI score postoperatively, they did not suffer from any clinical depression during the study period and depressive symptoms did not seem to play a significant role in coping with the disease.

### Strengths and limitations

The generalization of the results of this study and the possibilities for statistical analysis is limited due to the small number of patients. When designing the study, emphasis was placed on comprehensive neurocognitive testing. However, this extensive testing prevented many patients from participating. This could contribute to a possible inclusion/selection bias, as particularly motivated patients and/or patients with better cognitive performance may have participated. However, the calculated t-values show that the pre- and postoperative test results were average compared to a normative population, indicating that the study population was representative. In the future, a financial incentive or an offer of special support if neurocognitive deficits or depression is detected may increase the motivation to participate. Despite the small number of patients, our results are comparable with larger studies [14, 22]. The prospective study design, the comprehensive test battery and the pre- and postoperative testing after an average of approx. 6 months allow a comprehensive insight into the postoperative development of neurocognitive functions. In addition, the aspects of symptoms of depression and disease coping in the context of neurocognitive function have not yet been adequately addressed in the literature.

### Interpretation

As can be expected, most of our analysed patients were females. The tumor locations and symptoms at presentation were comparable to previous studies [14]. The impact of tumor location on neurocognitive impairment is controversial and may depend on the region of compression and the type of test [6, 14]. There were no previous diseases that were expected to be directly associated with neurocognitive disorders. Four patients with seizures were treated with antiepileptic drugs (i.e. levetiracetam), which have not been shown to have a negative effect on neurocognition [13]. The period between the pre- and postoperative tests was at least 127 days, with the surgery in between. Therefore, we do not assume that the results of such an extensive test battery could be influenced by a learning effect, and patients should not have been negatively impacted by any immediate postoperative sequelae.

In contrast to the study of Liouta et al. [14] we could not show that tumor size had an impact on preoperative neurocognitive deficits compared to smaller tumors. This could be explained by the smaller cohort in our study and a different cut-off value for tumor size. However, there was a trend towards poorer neurocognitive performance in patients with PTBE, particularly in the areas of logical memory (WMS IV), information processing (N-back), and responsiveness under stress (DT). Postoperatively, they tended to improve more than patients without PTBE. These findings support the findings of Liouta’s study. Interestingly, the neurocognitive performance of patients with postoperative resolution of PTBE and in patients with WHO grade 1 meningiomas appeared to be better pre- and postoperatively. Therefore, we hypothesize that penetration of the pia mater leading to PTBE may result in more pronounced neurocognitive deficits before and after surgery. As a pathophysiological correlate Koizumi et al. [12] identified a relative hypoperfusion in the region of edema compared to normal brain tissue in single photon emission computed tomography (SPECT). Furthermore, the authors describe a reversible depression of central benzodiazepine receptor viability after decompression of the brain tissue by tumor removal. The postoperative improvement of neurocognitive functioning might contribute to the significantly improved QoL concerning energy/fatigue and social functioning, among other aspects [10]. In this context, our prospective study highlights the importance of neurocognitive function in meningioma patients and its impact on QoL. As there is no consensus on which outcome measures are important in meningioma trials [18], we advocate the routine inclusion of neurocognitive domains as outcome measures in meningioma trials.

The fact that depressive symptoms play only a minor role in this cohort is consistent with the data from Kasper et al. who described mild to severe symptoms of depression in 7% of 14 surgically treated meningioma patients [11]. However, 29% of patients suffered from mild to severe anxiety symptoms in their small cohort. Other studies showed controversial results regarding the role of depression in meningioma patients, which could be explained by the different timing of the assessments during the course of the disease [3, 31]. As there were no symptoms of depression either pre- or postoperatively, the clinical relevance of the improvement in BDI II values is unclear. These results are comparable with the findings of Zweckberger et al. who described stable symptoms of depression in 90% of 58 patients with surgically treated skull base meningiomas [33]. Nevertheless, symptoms of depression or disease coping patterns must always be assessed individually to identify patients who need to be offered psychiatric treatment. The complex interaction between neurocognition, neuropsychological aspects such as disease coping and depression symptoms as well as tumor-specific and neurophysiological aspects requires large-scale multicenter studies to recruit a large number of patients to allow subgroup analysis and correlation analysis. The goal should be the early diagnosis of patients at increased risk for postoperative neurocognitive disorders to provide appropriate postoperative support when needed.

## Conclusion

The results in the present study suggest that resection of intracranial meningiomas leads to an improvement in multiple neurocognitive domains and quality of life. There is a trend towards poorer preoperative neurocognitive functioning and greater postoperative improvement in patients with PTBE. In this cohort, depression appears to play a minor role in the context of neurocognitive functioning and disease coping.

## Data Availability

Sharing the data with the public is not part of the ethical vote of this study. However, we can provide anonymized data at the request of the reviewers.

## Abbreviations

ADHD attention: deficit hyperactivity disorder
BDI II: Beck depression index II
DT: determination test
FQCD: Freiburg Questionnaire on Coping with Disease
NBV: N-back verbal test
PTBE: peritumoral brain edema
QoL: quality of life
RWT: Regensbuger verbal fluency test
SF-36: short form-36 health survey
SPECT: single photon emission computed tomography
SPM: standard progressive matrices
WHO: world health organization
WAIS IV: Wechsler Adult Intelligence Scale IV
WMS IV: Wechsler Memory Scale IV
